# Fractional differential equation modeling of the HBV infection with time delay and logistic proliferation

**DOI:** 10.3389/fpubh.2022.1036901

**Published:** 2022-11-11

**Authors:** Deshun Sun, Jingxiang Liu, Xiuyun Su, Guoxian Pei

**Affiliations:** ^1^Intelligent Medical Innovation Center, Southern University of Science and Technology Hospital, Shenzhen, China; ^2^Shenzhen Key Laboratory of Tissue Engineering, Shenzhen Second People's Hospital (The First Hospital Affiliated to Shenzhen University, Health Science Center), Shenzhen, China; ^3^School of Marine Electrical Engineering, Dalian Maritime University, Dalian, China

**Keywords:** HBV model, time delay, fractional order, stability, Hopf bifurcation

## Abstract

In this article, a fractional-order differential equation model of HBV infection was proposed with a Caputo derivative, delayed immune response, and logistic proliferation. Initially, infection-free and infection equilibriums and the basic reproduction number were computed. Thereafter, the stability of the two equilibriums was analyzed based on the fractional Routh–Hurwitz stability criterion, and the results indicated that the stability will change if the time delay or fractional order changes. In addition, the sensitivity of the basic reproduction number was analyzed to find out the most sensitive parameter. Lastly, the theoretical analysis was verified by numerical simulations. The results showed that the time delay of immune response and fractional order can significantly affect the dynamic behavior in the HBV infection process. Therefore, it is necessary to consider time delay and fractional order in modeling HBV infection and studying its dynamics.

## Introduction

Hepatitis B virus (HBV) can attack the liver and cause both acute and chronic diseases and further lead to fibrosis, cirrhosis, or even cancer. It is estimated that 296 million people have chronic hepatitis B, and 1.5 million new infections are reported each year; 820 000 people died of hepatitis B infections in 2019 (1). Therefore, HBV has become a major public health problem affecting human health (2).

Mathematical modeling and analysis of infectious viruses help understand the infection mechanism and realize the disease progression (3–5). Furthermore, mathematical modeling can also provide new insights to find the key factors to treat infectious diseases (6). In 1996, the basic ordinary differential equation (ODE) model of HBV infection was established with uninfected cells, infected cells, and free viruses (7). This is an early mathematical model for studying the spread of viruses. As research progresses, the mathematical modeling of virus transmission has become more and more complicated. For instance, Peter et al. (3) established a deterministic ODE model with six compartments to study the transmission dynamics of measles and obtained the best fit using available data, which could help health workers in decision-making and policymakers to frame policies to eradicate the spread of measles in Nigeria. Mayowa et al. (5) divided the population into six classes and formulated a six-compartmental deterministic model to investigate the effect of vaccination on the dynamics of tuberculosis in a given population. All the aforementioned mathematical models are based on ordinary differential equations with bilinear incidence rate.

Subsequently, a large number of dynamic models were proposed to describe and analyze virus infection according to different biological mechanisms (8–13); for example, because hepatocytes have the ability to regenerate, the models are constrained by the number of healthy and infected hepatocytes. Li et al. (10) developed a logistic growth model of HBV. Moreover, in order to characterize the time of a body's immune response after the virus infection of target cells, time delay has been considered. Therefore, Zhang et al. (13) proposed a susceptible-vaccinated-exposed-infectious-removed (SVEIR) epidemic model with two time delays and constructed a Lyapunov function to discuss the asymptotic stability of the positive equilibrium point. Babasola et al. (14) modeled the spread of COVID-19 with a convex incidence rate incorporated with a time delay and proved that delay can destabilize the system and lead to periodic oscillation.

In recent years, a fractional derivative for describing memory, history, and heredity effects in modeling physical, chemical, financial, and biological systems has received increasing attention (15–28). For example, Diethelm (29) used a fractional-order model to simulate the dynamics of a dengue fever outbreak. The results showed that the simulation accuracy of the fractional-order model is much higher than that of the integer-order derivative. Gilberto et al. (30) proposed a fractional-order model to research the dynamics of influenza A (H1N1), and the results showed that the fractional-order model was in good agreement with real data. Similarly, Ogunrinde et al. (27) divided the population into five classes and proposed a fractional-order differential equation model to study COVID-19. The basic reproduction number was calculated by the spectral radius method, and the stability analysis of the model was carried out by constructing the Lyapunov function. Finally, the parameters were estimated by collected data, and the model can offer guidance to policymakers.

In addition to the mathematical modeling of fractional differential equations for the aforementioned infectious diseases, there are also many studies that use the fractional-order model to characterize the process of HBV infections (31–33). For example, Simelane and Dlamini (33) established a fractional-order HBV model with a saturated incidence rate by using the Caputo fractional derivatives. Then, the basic reproduction number was calculated, and the stability of the equilibriums was discussed. The simulation results demonstrated that the fractional-order model is more appropriate for modeling HBV transmission dynamics than the integer-order model. The time of HBV entry into the healthy liver cells and the production of new virus particles should be taken into account; therefore, Gao et al. (32) established a three-dimensional delayed fractional-order HBV model, which included healthy hepatocytes, infected hepatocytes, and free viruses, as follows:


(1) {D0CFtσ1x(t)=λ1σ1−μ1σ1x(t)−β1σ1x(t)v(t)+δ1σ1y(t),D0CFtσ1y(t)=β1σ1x(t)v(t)−(α1σ1+δ1σ1)y(t),D0CFtσ2v(t)=c1σ2y(t−τ)e−ρτ−γ1σ2v(t).

This model has not considered the cytotoxic T lymphocyte (CTL) and alanine aminotransferase (ALT) levels, which reflect the extent of liver damage. Therefore, the items of CTL and ALT will be considered in our established model.

However, until now, no study has been designed to analyze the dynamics of HBV involving logistic proliferation, time delay, and items of CTL and ALT by fractional-order differential equations. Motivated by the aforementioned discussion, we proposed a fractional-order differential equation model with time delay and logistic proliferation in order to better understand the transmission mechanism of HBV in the human body.

The remaining part of this article is organized as follows: Section Mathematical model deals with the formulation of the model. Section Equilibriums and the basic reproduction number discusses the infection-free and infection equilibriums and the basic reproduction number. Section Equilibriums and the basic reproduction number discusses the stability analysis of the two equilibriums and analyzes the sensitivity of the basic reproduction number. Section Numerical simulation gives an account of the numerical simulations of equilibriums and the Hopf bifurcation. Finally, Section Conclusion and discussion comprises the conclusion and discussion.

## Mathematical model

Therefore, based on our work (34), we proposed a fractional-order differential equation model with time delay and logistic proliferation as follows:


(2) $$\begin{array}{l} \{\begin{array}{l} \frac{d^{\alpha }x(t)}{dt}=\xi^{\alpha }+r^{\alpha }x(t)\left(1-\frac{x(t)+y(t)}{T_{\max^{\alpha }}}\right)-d^{\alpha }x(t)-b^{\alpha }x(t)v(t),\\ \frac{d^{\alpha }y(t)}{dt}=b^{\alpha }x(t)v(t)-a^{\alpha }y(t)-k_{1}^{\alpha }y(t-\tau )z(t-\tau ),\\ \frac{d^{\alpha }v(t)}{dt}=k^{\alpha }y(t)-\varepsilon^{\alpha }v(t)-k_{2}^{\alpha }y(t-\tau )z(t-\tau ),\\ \frac{d^{\alpha }z(t)}{dt}=k_{3}^{\alpha }y(t-\tau )z(t-\tau )-k_{4}^{\alpha }z(t),\\ \frac{d^{\alpha }w(t)}{dt}=k_{5}^{\alpha }+k_{6}^{\alpha }y(t)z(t)-k_{7}^{\alpha }w(t). \end{array} \end{array}$$

where the variables *x, y, v, z*, and *w* represent the uninfected cells, infected cells, viruses, CTL level, and ALT level, respectively; ξ and *r* are the production rate and proliferation rate of uninfected cells, respectively; *T*_max_ is the maximum hepatocyte count in the liver; *d* is the death rate of uninfected cells; *b* is the infection rate of uninfected cells to become infected cells; *a* is the death rate of infected cells; *k*_1_ represents the cure rate of infected cells by CTL; *k* and ε are the production rate and death rate of free viruses, respectively; *k*_2_ represents the clearance rate of free viruses by CTL; *k*_3_ and *k*_4_ are the production rate and death rate of CTL, respectively; *k*_5_ is the natural production rate of ALT; *k*_6_ is the production rate of ALT from infected cells; *k*_7_ is the death rate; τ is time delay with the order of α(0 < α ≤ 1); and $\frac{d^{\alpha }x\left(t\right)}{dt}$, $\frac{d^{\alpha }y\left(t\right)}{dt}$, $\frac{d^{\alpha }v\left(t\right)}{dt}$, $\frac{d^{\alpha }z\left(t\right)}{dt}$, and $\frac{d^{\alpha }w\left(t\right)}{dt}$ denote the Caputo fractional derivatives. Hence, $\frac{d^{\alpha }x\left(t\right)}{dt}$ is defined as follows:


(3) $$\begin{array}{l} \frac{d^{\alpha }x_{i}}{dt}=I^{n-\alpha }\frac{d^{n}x}{dt^{n}}=\frac{1}{\Gamma \left(n-\alpha \right)}\int_{0}^{t}{\left(t-s\right)}^{\left(n-\alpha -1\right)}x^{\left(n\right)}\left(s\right)ds \end{array}$$

where *n* − 1 < α < *n, n* ∈ ℕ and Γ(▪) is the gamma function. When 0 < α < 1,


(4) $$\begin{array}{l} \frac{d^{\alpha }x}{dt}=\frac{1}{\Gamma \left(1-\alpha \right)}\int_{0}^{t}\frac{x^{\prime }\left(s\right)}{{\left(t-s\right)}^{\alpha }}ds \end{array}$$

Based on the aforementioned model, the equilibriums and stability analysis are discussed in Section Equilibriums and the basic reproduction number.

## Equilibriums and the basic reproduction number

In the following paragraphs, the equilibriums and the basic reproduction number are discussed.

### Equilibriums

The method to compute the equilibrium is to set $\frac{d^{\alpha }x\left(t\right)}{dt}=0$, $\frac{d^{\alpha }y\left(t\right)}{dt}=0$, $\frac{d^{\alpha }v\left(t\right)}{dt}=0$, $\frac{d^{\alpha }z\left(t\right)}{dt}=0$, and $\frac{d^{\alpha }w\left(t\right)}{dt}=0$. Hence, we get the following equations:


(5) $$\begin{array}{l} \{\begin{array}{l} \xi^{\alpha }-d^{\alpha }x(t)+r^{\alpha }x(t)\left(1-\frac{x(t)+y(t)}{T_{\max^{\alpha }}}\right)-b^{\alpha }x(t)v(t)=0,\\ b^{\alpha }x(t)v(t)-a^{\alpha }y(t)-k_{1}^{\alpha }y(t-\tau )z(t-\tau )=0,\\ k^{\alpha }y(t)-\varepsilon^{\alpha }v(t)-k_{2}^{\alpha }y(t-\tau )z(t-\tau )=0,\\ k_{3}^{\alpha }y(t-\tau )z(t-\tau )-k_{4}^{\alpha }z(t)=0,\\ k_{5}^{\alpha }+k_{6}^{\alpha }y(t)z(t)-k_{7}^{\alpha }w(t)=0. \end{array} \end{array}$$

The infection-free equilibrium ***E***_**0**_ denotes *x* ≠ 0, *w* ≠ 0, *y* = *v* = *z* = 0; thus, the infection-free equilibrium is as follows:


$$\begin{array}{l} E_{0}=\left(x_{0},y_{0},v_{0},z_{0},w_{0}\right)=\\ \left(\frac{T_{\max}^{\alpha }}{2r^{\alpha }}\left[-\left(d^{\alpha }-r^{\alpha }\right)+\sqrt{{\left(d^{\alpha }-r^{\alpha }\right)}^{2}+\frac{4\xi^{\alpha }r^{\alpha }}{T_{\max}^{\alpha }}}\right],0,0,0,\frac{k_{5}^{\alpha }}{k_{7}^{\alpha }}\right) \end{array}$$

Similarly, the infection equilibrium *E*_1_, which denotes *x* ≠ 0, *y* ≠ 0, *v* ≠ 0, *z* ≠ 0, *w* ≠ 0, was computed by the following equations:


(6) $$\begin{array}{l} \{\begin{array}{l} \xi^{\alpha }-d^{\alpha }x^{*}+r^{\alpha }x^{*}\left(1-\frac{x^{*}+y^{*}}{T_{\max^{\alpha }}}\right)-b^{\alpha }xv=0,\\ b^{\alpha }x^{*}v^{*}-a^{\alpha }y^{*}-k_{1}^{\alpha }y^{*}z^{*}=0,\\ k^{\alpha }y^{*}-\varepsilon^{\alpha }v^{*}-k_{2}^{\alpha }y^{*}z^{*}=0,\\ k_{3}^{\alpha }y^{*}z^{*}-k_{4}^{\alpha }z^{*}=0,\\ k_{5}^{\alpha }+k_{6}^{\alpha }y^{*}z^{*}-k_{7}^{\alpha }w^{*}=0. \end{array} \end{array}$$

The previous equations were solved, and the infection equilibrium was obtained as follows:

$x^{*}=-\frac{B}{3A}+\sqrt[{3}]{-\frac{q}{2}+\sqrt{\frac{q^{2}}{4}+\frac{p^{3}}{27}}}+\sqrt[{3}]{-\frac{q}{2}-\sqrt{\frac{q^{2}}{4}+\frac{p^{3}}{27}}},y^{*}\text{=}\frac{k_{4}^{\alpha }}{k_{3}^{\alpha }},v^{*}\text{=}\frac{a^{\alpha }k_{2}^{\alpha }k_{4}^{\alpha }+k^{\alpha }k_{1}^{\alpha }k_{4}^{\alpha }}{b^{\alpha }k_{2}^{\alpha }k_{3}^{\alpha }x^{*}+\varepsilon^{\alpha }k_{1}^{\alpha }k_{3}^{\alpha }},z^{*}=\frac{k^{\alpha }}{k_{2}^{\alpha }}-\frac{\varepsilon^{\alpha }\left(a^{\alpha }k_{2}^{\alpha }-k^{\alpha }k_{1}^{\alpha }\right)}{k_{2}^{\alpha }\left(b^{\alpha }k_{2}^{\alpha }x^{*}+\varepsilon^{\alpha }k_{1}^{\alpha }\right)},w^{*}=\frac{k_{5}^{\alpha }}{k_{7}^{\alpha }}+\frac{k^{\alpha }k_{4}^{\alpha }k_{6}^{\alpha }}{k_{2}^{\alpha }k_{3}^{\alpha }k_{7}^{\alpha }}-\frac{\varepsilon^{\alpha }k_{4}^{\alpha }k_{6}^{\alpha }\left(a^{\alpha }k_{2}^{\alpha }-k^{\alpha }k_{1}^{\alpha }\right)}{k_{2}^{\alpha }k_{3}^{\alpha }k_{7}^{\alpha }\left(b^{\alpha }k_{2}^{\alpha }x^{*}+\varepsilon^{\alpha }k_{1}^{\alpha }\right)}$.

where


$$\begin{array}{l} \begin{array}{c} A=\frac{b^{\alpha }r^{\alpha }k_{2}^{\alpha }}{T_{\max}^{\alpha }},B=-\left[b^{\alpha }k_{2}^{\alpha }\left(r^{\alpha }-d^{\alpha }-\frac{r^{\alpha }k_{4}^{\alpha }}{T_{\max}^{\alpha }k_{3}^{\alpha }}\right)-\frac{\varepsilon^{\alpha }r^{\alpha }k_{1}^{\alpha }}{T_{\max}^{\alpha }}\right],\\ C=-\left[b^{\alpha }\xi^{\alpha }k_{2}^{\alpha }+\varepsilon^{\alpha }k_{1}^{\alpha }\left(r^{\alpha }-d^{\alpha }-\frac{r^{\alpha }k_{4}^{\alpha }}{T_{\max}^{\alpha }k_{3}^{\alpha }}\right)-\frac{b^{\alpha }k_{4}^{\alpha }\left(a^{\alpha }k_{2}^{\alpha }+k^{\alpha }k_{1}^{\alpha }\right)}{k_{3}^{\alpha }}\right],\\ D=-\varepsilon^{\alpha }\xi^{\alpha }k_{1}^{\alpha },p=\frac{3AC-B^{2}}{3A^{2}},q=\frac{27A^{2}D-9ABC+2B^{3}}{27A^{3}}. \end{array} \end{array}$$

Thus, the infection equilibrium is as follows:


$$\begin{array}{l} E_{1}=(x^{*},y^{*},v^{*},z^{*},w^{*})\\ =(x^{*},\frac{k_{4}^{\alpha }}{k_{3}^{\alpha }},\frac{a^{\alpha }k_{2}^{\alpha }k_{4}^{\alpha }+k^{\alpha }k_{1}^{\alpha }k_{4}^{\alpha }}{b^{\alpha }k_{2}^{\alpha }k_{3}^{\alpha }x^{*}+\varepsilon^{\alpha }k_{1}^{\alpha }k_{3}^{\alpha }},\frac{k^{\alpha }}{k_{2}^{\alpha }}-\frac{\varepsilon^{\alpha }(a^{\alpha }k_{2}^{\alpha }-k^{\alpha }k_{1}^{\alpha })}{k_{2}^{\alpha }(b^{\alpha }k_{2}^{\alpha }x^{*}+\varepsilon^{\alpha }k_{1}^{\alpha })},\frac{k_{5}^{\alpha }}{k_{7}^{\alpha }}\\ +\frac{k^{\alpha }k_{4}^{\alpha }k_{6}^{\alpha }}{k_{2}^{\alpha }k_{3}^{\alpha }k_{7}^{\alpha }}-\frac{\varepsilon^{\alpha }k_{4}^{\alpha }k_{6}^{\alpha }(a^{\alpha }k_{2}^{\alpha }-k^{\alpha }k_{1}^{\alpha })}{k_{2}^{\alpha }k_{3}^{\alpha }k_{7}^{\alpha }(b^{\alpha }k_{2}^{\alpha }x^{*}+\varepsilon^{\alpha }k_{1}^{\alpha })}) \end{array}$$

### Basic reproduction number

The basic reproduction number can be calculated by the method of integral operator spectral radius given as follows:


$$\begin{array}{l} R_{0}=\rho \left(FV^{-1}\right) \end{array}$$

Thus, the basic reproduction number of *E*_0_ is as follows:


$$\begin{array}{l} R_{0}=\frac{b^{\alpha }k^{\alpha }x_{0}}{a^{\alpha }\varepsilon^{\alpha }}, \end{array}$$

where

$F=\left[\begin{array}{ccc} b^{\alpha }v & 0 & b^{\alpha }x\\ 0 & k^{\alpha }y & 0 \end{array}\right],V=\left[\begin{array}{ccc} 0 & a^{\alpha }+k_{1}^{\alpha }ze^{-\lambda \tau } & 0\\ 0 & k_{2}^{\alpha }ze^{-\lambda \tau } & \varepsilon^{\alpha } \end{array}\right].$

Similarly, the basic reproduction number of *E*_1_ is as follows:


$$\begin{array}{l} R_{1}=\frac{b^{\alpha }k^{\alpha }x^{*}}{\varepsilon^{\alpha }\left(a^{\alpha }+\frac{k^{\alpha }k_{1}^{\alpha }}{k_{2}^{\alpha }}-\frac{\varepsilon^{\alpha }k_{1}^{\alpha }\left(a^{\alpha }k_{2}^{\alpha }-k^{\alpha }k_{1}^{\alpha }\right)}{k_{2}^{\alpha }\left(b^{\alpha }k_{2}^{\alpha }x^{*}+\varepsilon^{\alpha }k_{1}^{\alpha }\right)}\right)} \end{array}$$

## Stability and sensitivity analyses

The local asymptotic stability of *E*_0_ and *E*_1_ is discussed in this part.

First, the Jacobi matrix was computed as follows:


(7) $$\begin{array}{l} Jac=\left[\begin{array}{ccccc} -d^{\alpha }+r^{\alpha }-\frac{2r^{\alpha }x+y}{T_{\max}^{\alpha }} & -\frac{r^{\alpha }x}{T_{\max}^{\alpha }} & -b^{\alpha }x & 0 & 0\\ b^{\alpha }v & -a^{\alpha }-k_{1}^{\alpha }ze^{-S^{\alpha }\tau } & b^{\alpha }x & -k_{1}^{\alpha }ye^{-S^{\alpha }\tau } & 0\\ 0 & k^{\alpha }-k_{2}^{\alpha }ze^{-S^{\alpha }\tau } & -\varepsilon^{\alpha } & -k_{2}^{\alpha }ye^{-S^{\alpha }\tau } & 0\\ 0 & k_{3}^{\alpha }ze^{-S^{\alpha }\tau } & 0 & k_{3}^{\alpha }ye^{-S^{\alpha }\tau }-k_{4}^{\alpha } & 0\\ 0 & k_{6}^{\alpha }z & 0 & k_{6}^{\alpha }y & -k_{7}^{\alpha } \end{array}\right] \end{array}$$

Based on the previous Jacobi matrix, we got the characteristic determinant:


$$\begin{array}{l} \left|S^{\alpha }I-Jac\right|\\ =\left|\begin{array}{ccccc} S^{\alpha }+d^{\alpha }-r^{\alpha }+\frac{2r^{\alpha }x+y}{T_{\max}^{\alpha }} & \frac{r^{\alpha }x}{T_{\max}^{\alpha }} & b^{\alpha }x & 0 & 0\\ -b^{\alpha }v & S^{\alpha }+a^{\alpha }+k_{1}^{\alpha }ze^{-S^{\alpha }\tau } & -b^{\alpha }x & k_{1}^{\alpha }ye^{-S^{\alpha }\tau } & 0\\ 0 & -k^{\alpha }+k_{2}^{\alpha }ze^{-S^{\alpha }\tau } & S^{\alpha }+\varepsilon^{\alpha } & k_{2}^{\alpha }ye^{-S^{\alpha }\tau } & 0\\ 0 & -k_{3}^{\alpha }ze^{-S^{\alpha }\tau } & 0 & S^{\alpha }+k_{4}^{\alpha }-k_{3}^{\alpha }ye^{-S^{\alpha }\tau } & 0\\ 0 & -k_{6}^{\alpha }z & 0 & -k_{6}^{\alpha }y & S^{\alpha }+k_{7}^{\alpha } \end{array}\right| \end{array}$$

Let *S*^α^ = λ, then the simplified characteristic determinant is as follows:


$$\begin{array}{l} |\lambda I-Jac|=\left|\begin{array}{ccccc} \lambda +d^{\alpha }-r^{\alpha }+\frac{2r^{\alpha }x+y}{T_{\max}} & \frac{r^{\alpha }x}{T_{\max}} & b^{\alpha }x & 0 & 0\\ -b^{\alpha }v & \lambda +a^{\alpha }+k_{1}^{\alpha }ze^{-\lambda \tau } & -b^{\alpha }x & k_{1}^{\alpha }ye^{-\lambda \tau } & 0\\ 0 & -k^{\alpha }+k_{2}^{\alpha }ze^{-\lambda \tau } & \lambda +\varepsilon^{\alpha } & k_{2}^{\alpha }ye^{-\lambda \tau } & 0\\ 0 & -k_{3}^{\alpha }ze^{-\lambda \tau } & 0 & \lambda +k_{4}^{\alpha }-k_{3}^{\alpha }ye^{-\lambda \tau } & 0\\ 0 & -k_{6}^{\alpha }z & 0 & -k_{6}^{\alpha }y & \lambda +k_{7}^{\alpha } \end{array}\right| \end{array}$$

### Local asymptotic stability of the infection-free equilibrium

The characteristic determinant at the infection-free equilibrium (*E*_0_) is as follows:


$$\begin{array}{l} \left|\lambda I-Jac\right|=\left|\begin{array}{ccccc} \lambda +d^{\alpha }-r^{\alpha }+\frac{2r^{\alpha }x_{0}}{T_{\max^{\alpha }}} & \frac{r^{\alpha }x_{0}}{T_{\max^{\alpha }}} & b^{\alpha }x & 0 & 0\\ 0 & \lambda +a^{\alpha } & -b^{\alpha }x_{0} & 0 & 0\\ 0 & -k^{\alpha } & \lambda +\varepsilon^{\alpha } & 0 & 0\\ 0 & 0 & 0 & \lambda +k_{4}^{\alpha } & 0\\ 0 & 0 & 0 & 0 & \lambda +k_{7}^{\alpha } \end{array}\right|\\ \;=(\lambda +d^{\alpha }-r^{\alpha }+\frac{2r^{\alpha }x_{0}}{T_{\max^{\alpha }}})(\lambda +k_{4}^{\alpha })(\lambda +k_{7}^{\alpha })[\left(\lambda +a^{\alpha })(\lambda +\varepsilon^{\alpha }\right)\\ \;-b^{\alpha }k^{\alpha }x_{0}] \end{array}$$

When |λ*I* − *Jac*| = 0, the eigenvalues are $\lambda_{1}=-d^{\alpha }+r^{\alpha }-\frac{2r^{\alpha }x_{0}}{T_{\max}^{\alpha }}$, $\lambda_{2}=k_{4}^{\alpha }$, $\lambda_{3}=k_{7}^{\alpha }$, $\lambda_{4}=\frac{-\left(a^{\alpha }+\varepsilon^{\alpha }\right)+\sqrt{{\left(a^{\alpha }+\varepsilon^{\alpha }\right)}^{2}-4\left(a^{\alpha }\varepsilon^{\alpha }-b^{\alpha }k^{\alpha }x_{0}\right)}}{2}$, and $\lambda_{5}=\frac{-\left(a^{\alpha }+\varepsilon^{\alpha }\right)-\sqrt{{\left(a^{\alpha }+\varepsilon^{\alpha }\right)}^{2}-4\left(a^{\alpha }\varepsilon^{\alpha }-b^{\alpha }k^{\alpha }x_{0}\right)}}{2}$.

Since *d* > *r* and $R_{0}=\frac{b^{\alpha }k^{\alpha }x_{0}}{a^{\alpha }\varepsilon^{\alpha }}<1$, we have λ_1,2,3,4,5_ < 0. Thus, $|\arg\left(S_{1,2,3,4,5}\right)|>\frac{\alpha \pi }{2}$.

Thus, we get the conclusion that when $R_{0}=\frac{b^{\alpha }k^{\alpha }x_{0}}{a^{\alpha }\varepsilon^{\alpha }}<1$, *E*_0_ is locally asymptotically stable.

### Local asymptotic stability of the infection equilibrium

The characteristic determinant at the infection equilibrium (*E*_1_) is as follows:


$$\begin{array}{l} \left|\begin{array}{ccccc} \lambda +d^{\alpha }-r^{\alpha }+\frac{2r^{\alpha }x+y}{T_{\max}^{\alpha }} & \frac{r^{\alpha }x}{T_{\max}^{\alpha }} & b^{\alpha }x & 0 & 0\\ -b^{\alpha }v & \lambda +a^{\alpha }+k_{1}^{\alpha }ze^{-\lambda \tau } & -b^{\alpha }x & k_{1}^{\alpha }ye^{-\lambda \tau } & 0\\ 0 & -k^{\alpha }+k_{2}^{\alpha }ze^{-\lambda \tau } & \lambda +\varepsilon^{\alpha } & k_{2}^{\alpha }ye^{-\lambda \tau } & 0\\ 0 & -k_{3}^{\alpha }ze^{-\lambda \tau } & 0 & \lambda +k_{4}^{\alpha }-k_{3}^{\alpha }ye^{-\lambda \tau } & 0\\ 0 & -k_{6}^{\alpha }z & 0 & -k_{6}^{\alpha }y & \lambda +k_{7}^{\alpha } \end{array}\right| \end{array}$$

$$\begin{array}{l} =\left(\lambda +k_{7}^{\alpha }\right)\left\{\begin{array}{c} \left(\lambda +A_{0}\right)\left[\begin{array}{c} \left(\lambda^{2}+\left(a^{\alpha }+\varepsilon^{\alpha }\right)\lambda +a^{\alpha }\varepsilon^{\alpha }+k_{1}^{\alpha }z\lambda e^{-\lambda \tau }+\varepsilon^{\alpha }k_{1}^{\alpha }ze^{-\lambda \tau }\right)\left(\lambda +k_{4}^{\alpha }-k_{3}^{\alpha }ye^{-\lambda \tau }\right)\\ +b^{\alpha }x\left(-k^{\alpha }+k_{2}^{\alpha }ze^{-\lambda \tau }\right)\left(\lambda +k_{4}^{\alpha }-k_{3}^{\alpha }ye^{-\lambda \tau }\right)+k_{1}^{\alpha }k_{3}^{\alpha }yz\left(\lambda +\varepsilon^{\alpha }\right)e^{-2\lambda \tau }\\ +b^{\alpha }k_{2}^{\alpha }xye^{-\lambda \tau } \end{array}\right]\\ +b^{\alpha }v\left[\frac{r\alpha x}{T_{\max}^{\alpha }}\left(\lambda +\varepsilon^{\alpha }\right)\left(\lambda +k_{4}^{\alpha }-k_{3}^{\alpha }ye^{-\lambda \tau }\right)-b^{\alpha }x\left(\begin{array}{c} \left(\lambda +k_{4}^{\alpha }-k_{3}^{\alpha }ye^{-\lambda \tau }\right)\left(k_{2}^{\alpha }ze^{-\lambda \tau }-k^{\alpha }\right)\\ +k_{2}^{\alpha }k_{3}^{\alpha }yze^{-2\lambda \tau } \end{array}\right)\right] \end{array}\right\} \end{array}$$

where $A_{0}=d^{\alpha }-r^{\alpha }+\frac{2r^{\alpha }x+y}{T_{\max}^{\alpha }}$.

For convenience, we made the following simplifications:


$$\begin{array}{l} \begin{array}{c} \left(\lambda^{2}+\left(a^{\alpha }+\varepsilon^{\alpha }\right)\lambda +a^{\alpha }\varepsilon^{\alpha }+k_{1}^{\alpha }z\lambda e^{-\lambda \tau }+\varepsilon^{\alpha }k_{1}^{\alpha }ze^{-\lambda \tau }\right)\left(\lambda +k_{4}^{\alpha }-k_{3}^{\alpha }ye^{-\lambda \tau }\right)\\ =\lambda^{3}+A_{1}\lambda^{2}+A_{2}\lambda +A_{3}+A_{4}\lambda^{2}e^{-\lambda \tau }+A_{5}\lambda e^{-\lambda \tau }+A_{6}e^{-\lambda \tau }+A_{7}\lambda e^{-2\lambda \tau }+A_{8}e^{-2\lambda \tau } \end{array} \end{array}$$

where


$$\begin{array}{l} \begin{array}{c} A_{1}=a^{\alpha }+\varepsilon^{\alpha }+k_{4}^{\alpha },A_{2}=a^{\alpha }\varepsilon^{\alpha }+\left(a^{\alpha }+\varepsilon^{\alpha }\right)k_{4}^{\alpha },A_{3}=a^{\alpha }\varepsilon^{\alpha }k_{4}^{\alpha },A_{4}=\left(k_{1}^{\alpha }z-k_{3}^{\alpha }y\right),\\ A_{5}=\varepsilon^{\alpha }k_{1}^{\alpha }z+k_{1}^{\alpha }k_{4}^{\alpha }z-\left(a^{\alpha }+\varepsilon^{\alpha }\right)k_{3}^{\alpha }y,A_{6}=\varepsilon^{\alpha }k_{1}^{\alpha }k_{4}^{\alpha }z-a^{\alpha }\varepsilon^{\alpha }k_{3}^{\alpha }y,A_{7}=-k_{1}^{\alpha }k_{3}^{\alpha }yz,\\ A_{8}=-\varepsilon^{\alpha }k_{1}^{\alpha }k_{3}^{\alpha }yz. \end{array} \end{array}$$

$$\begin{array}{l} \begin{array}{c} b^{\alpha }x\left(\lambda +k_{4}^{\alpha }-k_{3}^{\alpha }ye^{-\lambda \tau }\right)\left(-k^{\alpha }+k_{2}^{\alpha }ze^{-\lambda \tau }\right)+k_{1}^{\alpha }k_{3}^{\alpha }yz{\left(\lambda +\varepsilon \right)}^{\alpha }e^{-2\lambda \tau }+b^{\alpha }k_{2}^{\alpha }xye^{-\lambda \tau }\\ =A_{9}\lambda +A_{10}+A_{11}\lambda e^{-\lambda \tau }+A_{12}e^{-\lambda \tau }+A_{13}\lambda e^{-2\lambda \tau }+A_{14}e^{-2\lambda \tau } \end{array} \end{array}$$

where


$$\begin{array}{l} \begin{array}{c} A_{9}=-b^{\alpha }k^{\alpha }x,A_{10}=-b^{\alpha }k^{\alpha }k_{4}^{\alpha }x,A_{11}=b^{\alpha }k_{2}^{\alpha }xz,A_{12}=b^{\alpha }k^{\alpha }k_{3}^{\alpha }xy+b^{\alpha }k_{2}^{\alpha }k_{4}^{\alpha }xz+b^{\alpha }k_{2}^{\alpha }xy,\\ A_{13}=k_{1}^{\alpha }k_{3}^{\alpha }yz,A_{14}=\varepsilon^{\alpha }k_{1}^{\alpha }k_{3}^{\alpha }yz-bk_{2}^{\alpha }k_{3}^{\alpha }xyz \end{array} \end{array}$$

$$\begin{array}{l} \begin{array}{c} b^{\alpha }v\left[\frac{r^{\alpha }x}{T_{\max}^{\alpha }}\left(\lambda +\varepsilon^{\alpha }\right)\left(\lambda +k_{4}^{\alpha }-k_{3}^{\alpha }ye^{-\lambda \tau }\right)-b^{\alpha }x\left(\begin{array}{c} \left(\lambda +k_{4}^{\alpha }-k_{3}^{\alpha }ye^{-\lambda \tau }\right)\left(k_{2}^{\alpha }ze^{-\lambda \tau }-k^{\alpha }\right)\\ +k_{2}^{\alpha }k_{3}^{\alpha }yze^{-2\lambda \tau } \end{array}\right)\right]\\ =A_{15}\lambda^{2}+A_{16}\lambda +A_{17}+A_{18}\lambda e^{-\lambda \tau }+A_{19}e^{-\lambda \tau }+A_{20}e^{-2\lambda \tau } \end{array} \end{array}$$

where


$$A_{15}=\frac{b^{\alpha }r^{\alpha }xv}{T_{\max}^{\alpha }},A_{16}=b^{\alpha }v\left(\frac{r^{\alpha }k_{4}^{\alpha }x}{T_{\max}^{\alpha }}+\frac{\varepsilon^{\alpha }r^{\alpha }x}{T_{\max}}+b^{\alpha }k^{\alpha }x\right),A_{17}=b^{\alpha }v\left(\frac{\varepsilon^{\alpha }r^{\alpha }k_{4}^{\alpha }x}{T_{\max}^{\alpha }}+b^{\alpha }k^{\alpha }k_{4}^{\alpha }x\right),$$

$$\begin{array}{c} A_{18}=-b^{\alpha }v\left(\frac{r^{\alpha }k_{3}^{\alpha }xy}{T_{\max}^{\alpha }}+b^{\alpha }k_{2}^{\alpha }xz\right),A_{19}=-b^{\alpha }v\left(\frac{\varepsilon^{\alpha }r^{\alpha }k_{3}^{\alpha }xy}{T_{\max}^{\alpha }}+b^{\alpha }k_{2}^{\alpha }k_{4}^{\alpha }xz+b^{\alpha }k^{\alpha }k_{3}^{\alpha }xy\right),\\ A_{20}=-b^{2\alpha }xv\left(k_{2}^{\alpha }k_{3}^{\alpha }yz-k_{2}^{\alpha }k_{3}^{\alpha }yz\right). \end{array}$$

Thus, the characteristic determinant becomes as follows:


$$\begin{array}{l} \left(\lambda +k_{7}\right)\left\{\begin{array}{c} \left(\lambda +A_{0}\right)\left[\begin{array}{c} \lambda^{3}+A_{1}\lambda^{2}+A_{2}\lambda +A_{3}+A_{4}\lambda^{2}e^{-\lambda \tau }+A_{5}\lambda e^{-\lambda \tau }+A_{6}e^{-\lambda \tau }+A_{7}\lambda e^{-2\lambda \tau }\\ +A_{8}e^{-2\lambda \tau }+A_{9}\lambda +A_{10}+A_{11}\lambda e^{-\lambda \tau }+A_{12}e^{-\lambda \tau }+A_{13}\lambda e^{-2\lambda \tau }+A_{14}e^{-2\lambda \tau } \end{array}\right]\\ +A_{15}\lambda^{2}+A_{16}\lambda +A_{17}+A_{18}\lambda e^{-\lambda \tau }+A_{19}e^{-\lambda \tau }+A_{20}e^{-2\lambda \tau } \end{array}\right\} \end{array}$$

$$\begin{array}{l} =\left(\lambda +k_{7}^{\alpha }\right)\left\{\left[\begin{array}{c} \lambda^{4}+B_{1}\lambda^{3}+B_{2}\lambda^{2}+B_{3}\lambda +B_{4}+B_{5}\lambda^{3}e^{-\lambda \tau }+B_{6}\lambda^{2}e^{-\lambda \tau }+B_{7}\lambda e^{-\lambda \tau }+B_{8}e^{-\lambda \tau }\\ +B_{9}\lambda^{2}e^{-2\lambda \tau }+B_{10}\lambda e^{-2\lambda \tau }+B_{11}e^{-2\lambda \tau } \end{array}\right]\right\} \end{array}$$

where


$$\begin{array}{l} \begin{array}{c} B_{1}=A_{1}+A_{0},B_{2}=A_{2}+A_{9}+A_{0}A_{1}+A_{15},B_{3}=A_{3}+A_{10}+A_{0}A_{2}+A_{0}A_{9}+A_{16},\\ B_{4}=A_{0}A_{3}+A_{0}A_{10}+A_{17},B_{5}=A_{4},B_{6}=A_{5}+A_{11}+A_{0}A_{4},B_{7}=A_{6}+A_{12}+A_{0}A_{5}+A_{0}A_{11}+A_{18},\\ B_{8}=A_{0}A_{6}+A_{0}A_{12}+A_{19},B_{9}=A_{7}+A_{13},B_{10}=A_{8}+A_{14}+A_{0}A_{7}+A_{0}A_{13},B_{11}=A_{0}A_{8}+A_{0}A_{14}+A_{20}. \end{array} \end{array}$$

For further simplification, we derived the following assignment:


$$\begin{array}{l} \begin{array}{c} C_{1}=B_{1}\text{+}k_{7},C_{2}=B_{2}+k_{7}B_{1},C_{3}=B_{3}+k_{7}B_{2},C_{4}=B_{4}+k_{7}B_{3},C_{5}=k_{7},C_{6}=B_{5},C_{7}=B_{6}+k_{7}B_{5},\\ C_{8}=B_{7}+k_{7}B_{6},C_{9}=B_{8}+k_{7}B_{7},C_{10}=k_{7}B_{8},C_{11}=B_{9},C_{12}=B_{10}+k_{7}B_{9},C_{13}=B_{11}+k_{7}B_{10},C_{14}=k_{7}B_{11}. \end{array} \end{array}$$

The characteristic determinant is as follows:


(8) $$\begin{array}{l} \begin{array}{c} H\left(\lambda ;\tau \right)\text{=}\lambda^{5}+C_{1}\lambda^{4}+C_{2}\lambda^{3}+C_{3}\lambda^{2}+C_{4}\lambda +C_{5}+\left(C_{6}\lambda^{4}+C_{7}\lambda^{3}+C_{8}\lambda^{2}+C_{9}\lambda +C_{10}\right)e^{-\lambda \tau }\\ +\left(C_{11}\lambda^{3}+C_{12}\lambda^{2}+C_{13}\lambda +C_{14}\right)e^{-2\lambda \tau }\text{=0} \end{array} \end{array}$$

When τ = 0, the previous equation becomes as follows:


(9) $$\begin{array}{l} \lambda^{5}+D_{1}\lambda^{4}+D_{2}\lambda^{3}+D_{3}\lambda^{2}+D_{4}\lambda +D_{5}=0 \end{array}$$

where


$$\begin{array}{l} D_{1}=C_{1}+C_{6},D_{2}=C_{2}+C_{7}+C_{11},D_{3}=C_{3}+C_{8}+C_{12},D_{4}=C_{4}+C_{9}+C_{13},D_{5}=C_{5}+C_{10}+C_{14}. \end{array}$$

Based on equation (9), we get the following lemma by applying the Routh–Hurwitz criterion.

**Lemma** If equation (9) satisfies Δ_1_ ≡ *D*_1_ > 0, $\Delta_{2}\equiv |\begin{array}{cc} D_{1} & 1\\ D_{3} & D_{2}\\ \; \end{array}|>0$, and $\Delta_{3}\equiv |\begin{array}{ccc} D_{1} & 1 & 0\\ D_{3} & D_{2} & D_{1}\\ D_{5} & D_{4} & D_{3}\\ \; \end{array}|>0$, *E*_1_ is locally asymptotically stable when τ = 0.

**Proof**. The detailed proof can be referred to Peter et al. (26), Ogunrinde et al. (27).

The aforementioned lemma indicated that when τ = 0, all roots of *H*(λ; τ) are to the left of the imaginary axis, and some roots may cross to the right from the imaginary axis as τ increases. Thus, *E*_1_ is unstable because of its positive real parts.

Then, the stability of system (2) was investigated when τ > 0.

Both sides of equation (8) were multiplied by *e*^λτ^:


(10) $$\begin{array}{l} \begin{array}{c} \left(C_{6}\lambda^{4}+C_{7}\lambda^{3}+C_{8}\lambda^{2}+C_{9}\lambda +C_{10}\right)+\left(\lambda^{5}+C_{1}\lambda^{4}+C_{2}\lambda^{3}+C_{3}\lambda^{2}+C_{4}\lambda +C_{5}\right)e^{\lambda \tau }\\ +\left(C_{11}\lambda^{3}+C_{12}\lambda^{2}+C_{13}\lambda +C_{14}\right)e^{-\lambda \tau }\text{=0} \end{array} \end{array}$$

Suppose the aforementioned equation has a purely imaginary root λ = *iω* (ω > 0), then we have *e*^*iω*^ = cosω+*i*sinω, *e*^−*iω*^ = cosω − *i*sinω. Substituting λ = *iω* into equation (10), we have


(11) $$\begin{array}{l} C_{6}\lambda^{4}+C_{7}\lambda^{3}+C_{8}\lambda^{2}+C_{9}\lambda +C_{10}=C_{6}\omega^{4}-C_{7}\omega^{3}i-C_{8}\omega^{2}+C_{9}\omega i+C_{10} \end{array}$$

(12) $$\begin{array}{l} (\lambda^{5}+C_{1}\lambda^{4}+C_{2}\lambda^{3}+C_{3}\lambda^{2}+C_{4}\lambda +C_{5})e^{\lambda \tau }=(C_{1}\omega^{4}-C_{3}\omega^{2}+C_{5})\cos\omega \tau +(\omega^{5}-\\ C_{2}\omega^{3}+C_{4}\omega )\cos\omega \tau i+(-\omega^{5}+C_{2}\omega^{3}-C_{4}\omega )\sin\omega \tau +(C_{1}\omega^{4}-C_{3}\omega^{2}+C_{5})\sin\omega \tau i \end{array}$$

(13) $$\begin{array}{l} \begin{array}{c} \left(C_{11}\lambda^{3}+C_{12}\lambda^{2}+C_{13}\lambda +C_{14}\right)e^{-\lambda \tau }=\left(-C_{12}\omega^{2}+C_{14}\right)\cos\omega \tau +\left(-C_{11}\omega^{3}+C_{13}\omega \right)\\ \times \cos\omega \tau i+\left(-C_{11}\omega^{3}+C_{13}\omega \right)\sin\omega \tau +\left(C_{12}\omega^{2}-C_{14}\right)\sin\omega \tau i \end{array} \end{array}$$

Therefore, equation (10) becomes as follows:


$$\begin{array}{l} \begin{array}{c} \left(C_{1}\omega^{4}-C_{3}\omega^{2}-C_{12}\omega^{2}+C_{5}+C_{14}\right)\cos\omega \tau +\left(\omega^{5}-C_{2}\omega^{3}-C_{11}\omega^{3}+C_{4}\omega +C_{13}\omega \right)\cos\omega \tau i\\ +\left(-\omega^{5}+C_{2}\omega^{3}-C_{11}\omega^{3}-C_{4}\omega +C_{13}\omega \right)\sin\omega \tau +\left(C_{1}\omega^{4}-C_{3}\omega^{2}+C_{12}\omega^{2}+C_{5}-C_{14}\right)\sin\omega \tau i\\ +C_{6}\omega^{4}-C_{7}\omega^{3}i-C_{8}\omega^{2}+C_{9}\omega i+C_{10}=0 \end{array} \end{array}$$

For convenience, we assumed the following:


$$\begin{array}{l} \begin{array}{c} a_{1}=C_{1}\omega^{4}-C_{3}\omega^{2}-C_{12}\omega^{2}+C_{5}+C_{14},\;a_{2}=\omega^{5}-C_{2}\omega^{3}-C_{11}\omega^{3}+C_{4}\omega +C_{13}\omega ,\\ a_{3}=-\omega^{5}+C_{2}\omega^{3}-C_{11}\omega^{3}-C_{4}\omega +C_{13}\omega ,\;a_{4}=C_{1}\omega^{4}-C_{3}\omega^{2}+C_{12}\omega^{2}+C_{5}-C_{14}. \end{array} \end{array}$$

Thus, we get


(14) $$\begin{array}{l} \begin{array}{c} a_{1}\cos\omega \tau +a_{2}\cos\omega \tau i+a_{3}\sin\omega \tau +a_{4}\sin\omega \tau i+C_{6}\omega^{4}-C_{7}\omega^{3}i-C_{8}\omega^{2}\\ +C_{9}\omega i+C_{10}=0 \end{array} \end{array}$$

The real part after separating the real and imaginary parts is as follows:


(15) $$\begin{array}{l} a_{1}\cos\omega \tau +a_{3}\sin\omega \tau =-C_{6}\omega^{4}+C_{8}\omega^{2}-C_{10}=D_{1} \end{array}$$

and the imaginary part is as follows:


(16) $$\begin{array}{l} a_{2}\cos\omega \tau +a_{4}\sin\omega \tau =C_{7}\omega^{3}-C_{9}\omega =D_{2} \end{array}$$

It follows from the real part and imaginary part that


(17) $$\begin{array}{l} \cos\omega \tau =\frac{a_{4}D_{1}-a_{3}D_{2}}{a_{1}a_{4}-a_{2}a_{3}};\sin\omega \tau =\frac{a_{1}D_{2}-a_{2}D_{1}}{a_{1}a_{4}-a_{2}a_{3}} \end{array}$$

Suppose equation (10) has ñ(1 ≤ ñ ≤ 5) positive real roots, denoted by *x*_*n*_(1 ≤ *n* ≤ ñ).

Let $\sqrt{x_{n}}=\omega$, we get


$$\begin{array}{l} \begin{array}{c} \cos\left(\sqrt{x_{n}}\tau \right)=Q_{n}\\ =\frac{\left(C_{1}\omega^{4}-C_{3}\omega^{2}+C_{12}\omega^{2}+C_{5}-C_{14}\right)\left(-C_{6}\omega^{4}+C_{8}\omega^{2}-C_{10}\right)-\left(-\omega^{5}+C_{2}\omega^{3}-C_{11}\omega^{3}-C_{4}\omega +C_{13}\omega \right)\left(C_{7}\omega^{3}-C_{9}\omega \right)}{\left(C_{1}\omega^{4}-C_{3}\omega^{2}-C_{12}\omega^{2}+C_{5}+C_{14}\right)\left(C_{1}\omega^{4}-C_{3}\omega^{2}+C_{12}\omega^{2}+C_{5}-C_{14}\right)-\left(\omega^{5}-C_{2}\omega^{3}-C_{11}\omega^{3}+C_{4}\omega +C_{13}\omega \right)\left(-\omega^{5}+C_{2}\omega^{3}-C_{11}\omega^{3}-C_{4}\omega +C_{13}\omega \right)} \end{array} \end{array}$$

$$\begin{array}{l} \begin{array}{c} \sin\left(\sqrt{x_{n}}\tau \right)=P_{n}\\ =\frac{\left(C_{1}\omega^{4}-C_{3}\omega^{2}-C_{12}\omega^{2}+C_{5}+C_{14}\right)\left(C_{7}\omega^{3}-C_{9}\omega \right)-\left(\omega^{5}-C_{2}\omega^{3}-C_{11}\omega^{3}+C_{4}\omega +C_{13}\omega \right)\left(-C_{6}\omega^{4}+C_{8}\omega^{2}-C_{10}\right)}{\left(C_{1}\omega^{4}-C_{3}\omega^{2}-C_{12}\omega^{2}+C_{5}+C_{14}\right)\left(C_{1}\omega^{4}-C_{3}\omega^{2}+C_{12}\omega^{2}+C_{5}-C_{14}\right)-\left(\omega^{5}-C_{2}\omega^{3}-C_{11}\omega^{3}+C_{4}\omega +C_{13}\omega \right)\left(-\omega^{5}+C_{2}\omega^{3}-C_{11}\omega^{3}-C_{4}\omega +C_{13}\omega \right)} \end{array} \end{array}$$

Let


$$\begin{array}{l} \tau_{n}^{\left(j\right)}=\{\begin{array}{ll} \frac{1}{\sqrt{x_{n}}}\left[\arccos(Q_{n})+2j\pi \right], & if\;P_{n}\geq 0\\ \frac{1}{\sqrt{x_{n}}}\left[2\pi -\arccos(Q_{n})+2j\pi \right], & if\;P_{n}<0 \end{array} \end{array}$$

Here, the positive integer *n* satisfies 1 ≤ *n* ≤ ñ, *j* = 0, 1, 2, ...

Thus, from the aforementioned equation, we know that the characteristic equation has a pair of purely imaginary roots $\pm i\sqrt{x_{n}}$. We define $\lambda_{n}^{\left(j\right)}\left(\tau \right)=\alpha_{n}^{\left(j\right)}\left(\tau \right)+i\omega_{n}^{\left(j\right)}\left(\tau \right)$ as the root of equation (10) near $\tau_{n}^{\left(j\right)}$ for every 1 ≤ *n* ≤ ñ and *j*, satisfying $\alpha_{n}^{\left(j\right)}\left(\tau_{n}^{\left(j\right)}\right)=0$ and $\omega_{n}^{\left(j\right)}\left(\tau_{n}^{\left(j\right)}\right)=\sqrt{x_{n}}$. In summary, we arrived at the following theorem:

**Theorem 1** When $\tau \in \left[0,\tau_{n_{0}}^{\left(0\right)}\right)$ and there are positive real roots in equation (10), infection equilibrium *E*_1_ is locally asymptotically stable, where


$$\begin{array}{l} \tau_{n_{0}}^{\left(0\right)}=\min\{\tau_{n}^{\left(j\right)}|1\leq n\leq ñ,j=0,1,2,...\}. \end{array}$$

**Proof**. When $\tau \in \left[0,\tau_{n_{0}}^{\left(0\right)}\right)$ and equation (10) have no positive real roots, where $\tau_{n_{0}}^{\left(0\right)}=\min\left\{\tau_{n}^{\left(j\right)}|1\leq n\leq ñ,j=0,1,2,...\right\}$, all the roots have strictly negative real parts. Thus, *E*_1_ is locally asymptotically stable for $\tau \in \left[0,\tau_{n_{0}}^{\left(0\right)}\right)$.

### Sensitivity of the basic reproduction number

In this part, the sensitivity index of the basic reproduction number is explored in order to find out the most sensitive parameter that can significantly affect the basic reproduction number and give proper treatment strategies (3).

The sensitivity index can be computed by using the following equation:


(18) $$\begin{array}{l} K_{q}^{R_{0}}=\frac{\partial R_{0}}{\partial q}\times \frac{q}{R_{0}} \end{array}$$

The basic reproduction number of *E*_0_ is as follows:


$$\begin{array}{l} R_{0}=\frac{b^{\alpha }k^{\alpha }x_{0}}{a^{\alpha }\varepsilon^{\alpha }},where\;x_{0}=\frac{T_{\max}^{\alpha }}{2r^{\alpha }}\left[-\left(d^{\alpha }-r^{\alpha }\right)+\sqrt{{\left(d^{\alpha }-r^{\alpha }\right)}^{2}+\frac{4\xi^{\alpha }r^{\alpha }}{T_{\max}^{\alpha }}}\right]. \end{array}$$

The results of sensitivity indexes (Table 1) demonstrated that the infection rate of uninfected cells to become infected cells (*b*), production rate of free viruses (*k*), maximum hepatocyte counts in the liver (*T*_max_), and production rate of uninfected cells (ξ) have the highest positive index. Therefore, decreasing the infection rate, the production rate of free viruses, and the production rate of uninfected cells can help treat patients with hepatitis B. On the contrary, the death rate of infected cells (*a*), the death rate of free viruses (ε), and the death rate of uninfected cells (*d*) have the highest negative index. This also suggests that increasing the death rate of infected cells, the death rate of free viruses, and the death rate of uninfected cells can also keep *R*_0_ < 1 and help the treatment of patients with hepatitis B.

**Table 1:** Sensitivity indexes of *R*_0_ to model parameters.

**Parameter**	**Sensitivity index**
*b*	0.9000
*k*	0.9000
*a*	−0.8100
ε	−0.8100
*T*_max_	0.9298
*d*	−0.8951
*r*	−0.4518
ξ	0.8716

## Numerical simulation

In this section, a simulation is carried out to prove the accuracy of the aforementioned theoretical analysis.

### Algorithm

Before the simulation, first, we provide the algorithm to solve the fractional-order differential equation (35, 36):


(19) $$\begin{array}{l} \{\begin{array}{l} x(t_{k})=\left[\xi^{\alpha }-d^{\alpha }x(t_{k-1})+r^{\alpha }x(t_{k-1})\left(1-\frac{x(t_{k-1})+y(t_{k-1})}{T_{\max^{\alpha }}}\right)-b^{\alpha }x(t_{k-1})v(t_{k-1})\right]h^{q_{1}}-\sum_{j=v}^{k}c_{j}^{\left(q_{1}\right)}x(t_{k-j}),\\ y(t_{k})=\left[b^{\alpha }x(t_{k-1})v(t_{k-1})-ay(t_{k-1})-k_{1}^{\alpha }y(t_{k-m-1})z(t_{k-m-1})\right]h^{q_{1}}-\sum_{j=v}^{k}c_{j}^{\left(q_{1}\right)}y(t_{k-j}),\\ v(t_{k})=\left[k^{\alpha }y(t_{k-1})-\varepsilon^{\alpha }v(t_{k-1})-k_{2}^{\alpha }y(t_{k-m-1})z(t_{k-m-1})\right]h^{q_{1}}-\sum_{j=v}^{k}c_{j}^{\left(q_{1}\right)}v(t_{k-j}),\\ z(t_{k})=\left[k_{3}^{\alpha }y(t_{k-m-1})z(t_{k-m-1})-k_{4}^{\alpha }z(t_{k-1})\right]h^{q_{1}}-\sum_{j=v}^{k}c_{j}^{\left(q_{1}\right)}z(t_{k-j}),\\ w(t_{k})=\left[k_{5}^{\alpha }+k_{6}^{\alpha }y(t_{k-1})z(t_{k-1})-k_{7}^{\alpha }w(t_{k-1})\right]h^{q_{1}}-\sum_{j=v}^{k}c_{j}^{\left(q_{1}\right)}w(t_{k-j}), \end{array} \end{array}$$

where *T*_*sim*_ is time length, *k* = 1, 2, 3, ..., *N*, *N* = [*T*_*sim*_/*h*], *m* = [τ/*h*], and *x*(0) = *x*_0_, *v*(0) = *v*_0_, *w*(0) = *w*_0_, *y*(*t*) = *y*_0_, *z*(*t*) = *z*_0_, *t* ∈ [−τ, 0] are the initial conditions. $c_{0}^{\left(q\right)}=1,c_{j}^{\left(q\right)}=\left(1-\frac{1+q}{j}\right)c_{j-1}^{\left(q\right)}$.

### Simulation of asymptotically stable infection-free equilibrium

First, we simulate the case of infection-free. The parameters are shown in Table 2.

**Table 2:** Description of parameters and values when *R*_0_ < 1.

**Parameter**	**Description**	**Value**	**Source**
ξ	Production rate of uninfected cells	4.6664	(37)
*d*	Death rate of uninfected cells	2.1897	Estimated
*r*	Proliferation rate of uninfected cells	0.0924	Estimated
*T*_max_	Maximum hepatocyte counts in the liver	4.2843	Estimated
*b*	Infection rate of uninfected cells to become infected cells	1.4042	Estimated
*a*	Death rate of infected cells	3.8707	Estimated
*k*_1_	Cure rate of infected cells by CTL	1.8838	(37)
*k*	Production rate of free viruses	1.3655	(37)
ε	Death rate of free viruses	1.48663	Estimated
*k*_2_	Clearance rate of free viruses by CTL	1.2661	Estimated
*k*_3_	Production rate of CTL	3.8549	(37)
*k*_4_	Death rate of CTL	1.1395	Estimated
*k*_5_	Natural production rate of ALT	1.8789	(37)
*k*_6_	Production rate of ALT from infected cells	0.12002	Estimated
*k*_7_	Death rate of ALT	1.2557	Estimated

The time length is 400, and the initial conditions are *x*(0) = 1, *v*(0) = 1, *w*(0) = 1, *y*(*t*) = 1, *z*(*t*) = 1, *t* ∈ [−τ, 0]. The order α = 0.9 and the time delay τ = 0.7. Therefore, we have *E*_0_ = (*x*_0_, *y*_0_, *v*_0_, *z*_0_, *w*_0_) = (2.0289, 0, 0, 0, 1.4372), and the basic reproduction number *R*_0_ = 0.7546.

**Figure 1:**
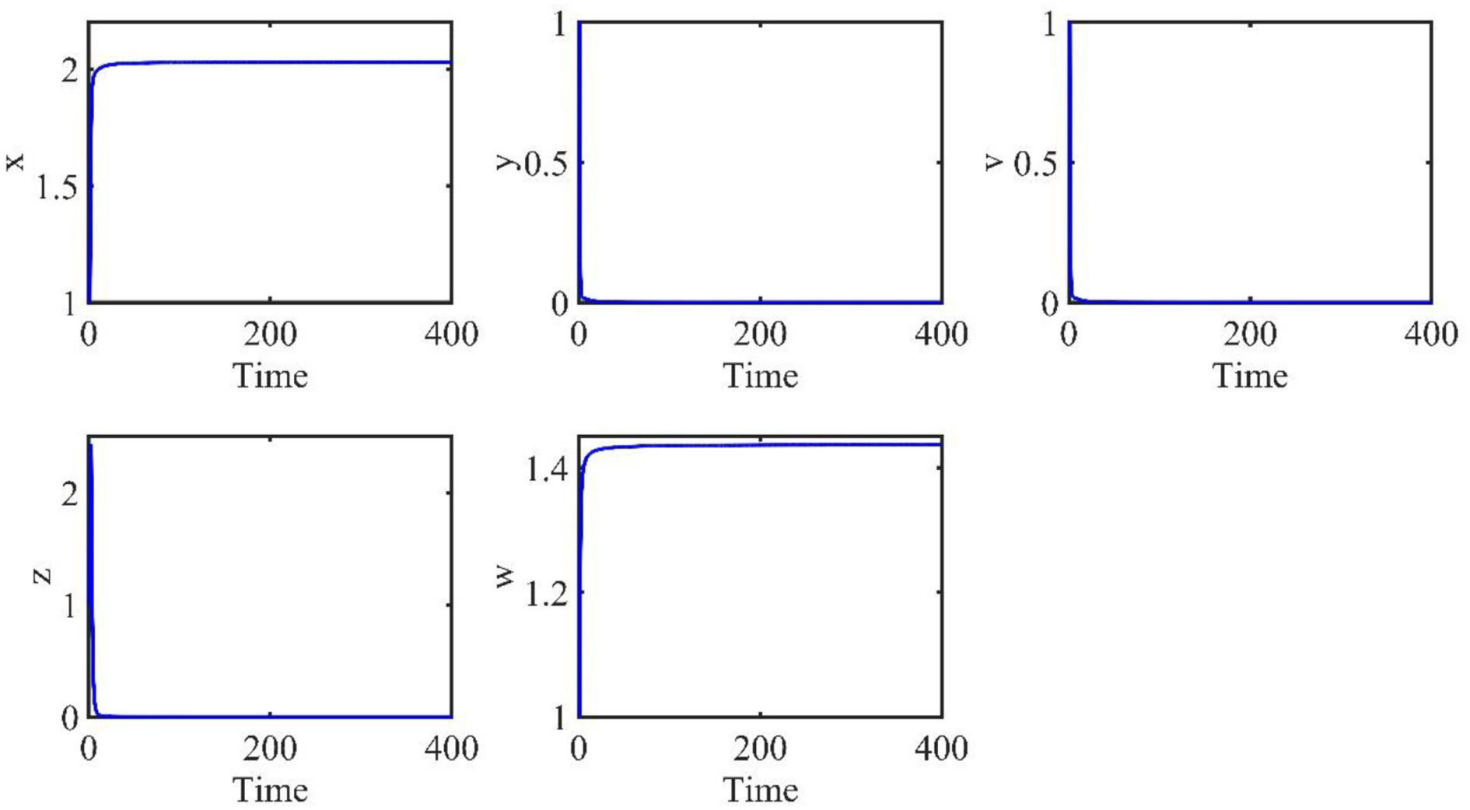
Dynamic change trend of uninfected cells (*x*), infected cells (*y*), free viruses (*v*), CTLs (*z*), and ALT (*w*) with *R*_0_ < 1 and τ = 0.7.

The behaviors of the uninfected cells (*x*), infected cells (*y*), free viruses (*v*), CTLs (*z*), and ALT (*w*) are shown in Figure 1. In Figure 1, all individuals converge to the infection-free equilibrium *E*_0_, and the basic reproduction number *R*_0_ is 0.7546, which is smaller than 1. This coincides with our theoretical analysis, which showed that when *R*_0_ < 1, the infection-free equilibrium *E*_0_ is asymptotically stable.

### Simulation of asymptotically stable infection equilibrium

The theoretical analysis of the infection equilibrium is verified in this section. Similarly, the parameters are shown in Table 3.

**Table 3:** Description of parameters and values when *R*_1_ > 1.

**Parameter**	**Description**	**Value**	**Source**
ξ	Production rate of uninfected cells	4.6664	(37)
*d*	Death rate of uninfected cells	2.1897	Estimated
*r*	Proliferation rate of uninfected cells	0.0924	Estimated
*T*_max_	Maximum hepatocyte counts in the liver	4.2843	Estimated
*b*	Infection rate of uninfected cells to become infected cells	2.4042	Estimated
*a*	Death rate of infected cells	2.8707	Estimated
*k*_1_	Cure rate of infected cells by CTL	1.8838	(37)
*k*	Production rate of free viruses	2.3655	(37)
ε	Death rate of free viruses	0.48663	(37)
*k*_2_	Clearance rate of free viruses by CTL	1.2661	Estimated
*k*_3_	Production rate of CTL	3.8549	(37)
*k*_4_	Death rate of CTL	1.1395	Estimated
*k*_5_	Natural production rate of ALT	1.8789	(37)
*k*_6_	Production rate of ALT from infected cells	0.12002	Estimated
*k*_7_	Death rate of ALT	1.2557	Estimated

The initial conditions are the same as in the previous section. The time length is 400. The order α = 0.9, and the time delay τ = 1.2. Therefore, the infection equilibrium is as follows: *E*_1_ = (*x*_1_, *y*_1_, *v*_1_, *z*_1_, *w*_1_) = (1.2762, 0.3341, 0.5338, 1.0787, 1.4802), and the basic reproduction number is *R*_1_ = 2.0132.

**Figure 2:**
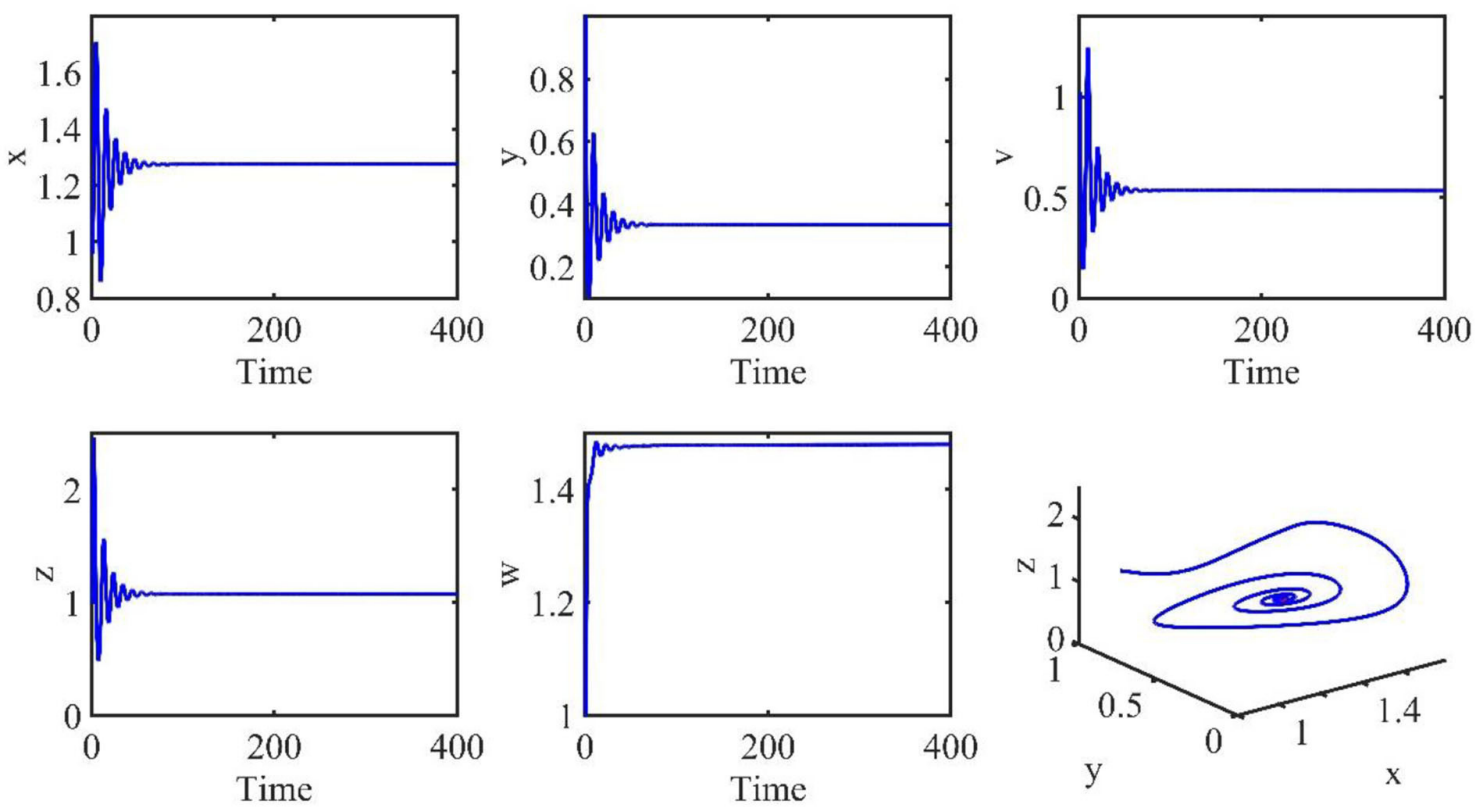
Dynamic change trend of uninfected cells (*x*), infected cells (*y*), free viruses (*v*), CTLs (*z*), ALT (*w*), and phase portraits of the xyz-space with *R*_1_ > 1 and τ = 1.2.

**Figure 3:**
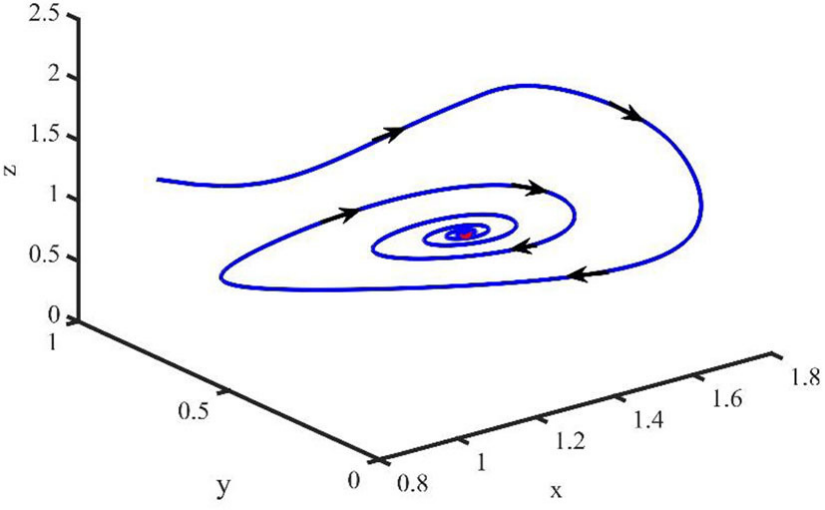
Phase portraits of the xyz-space with *R*_1_ > 1 and τ = 1.2.

Figure 2 is the behavior of the uninfected cells (*x*), infected cells (*y*), free viruses (*v*), CTLs (*z*), and ALT (*w*) with *R*_1_ > 1 and τ = 1.2. From Figure 2, we know that although all the individuals oscillate at the beginning, they converge to infection equilibrium *E*_1_shortly. Figure 3 shows the phase portraits of the uninfected cell–infected cell–free virus space; the arrow indicates the direction of convergence of the phase portraits, and it converges to the infection equilibrium *E*_1_ (red dot).

### Simulation of the Hopf bifurcation of the infection equilibrium

In this subsection, the Hopf bifurcation of the infection equilibrium is simulated. All parameters are the same as those in Section Simulation of asymptotically stable infection equilibrium, except τ = 3.2.

**Figure 4:**
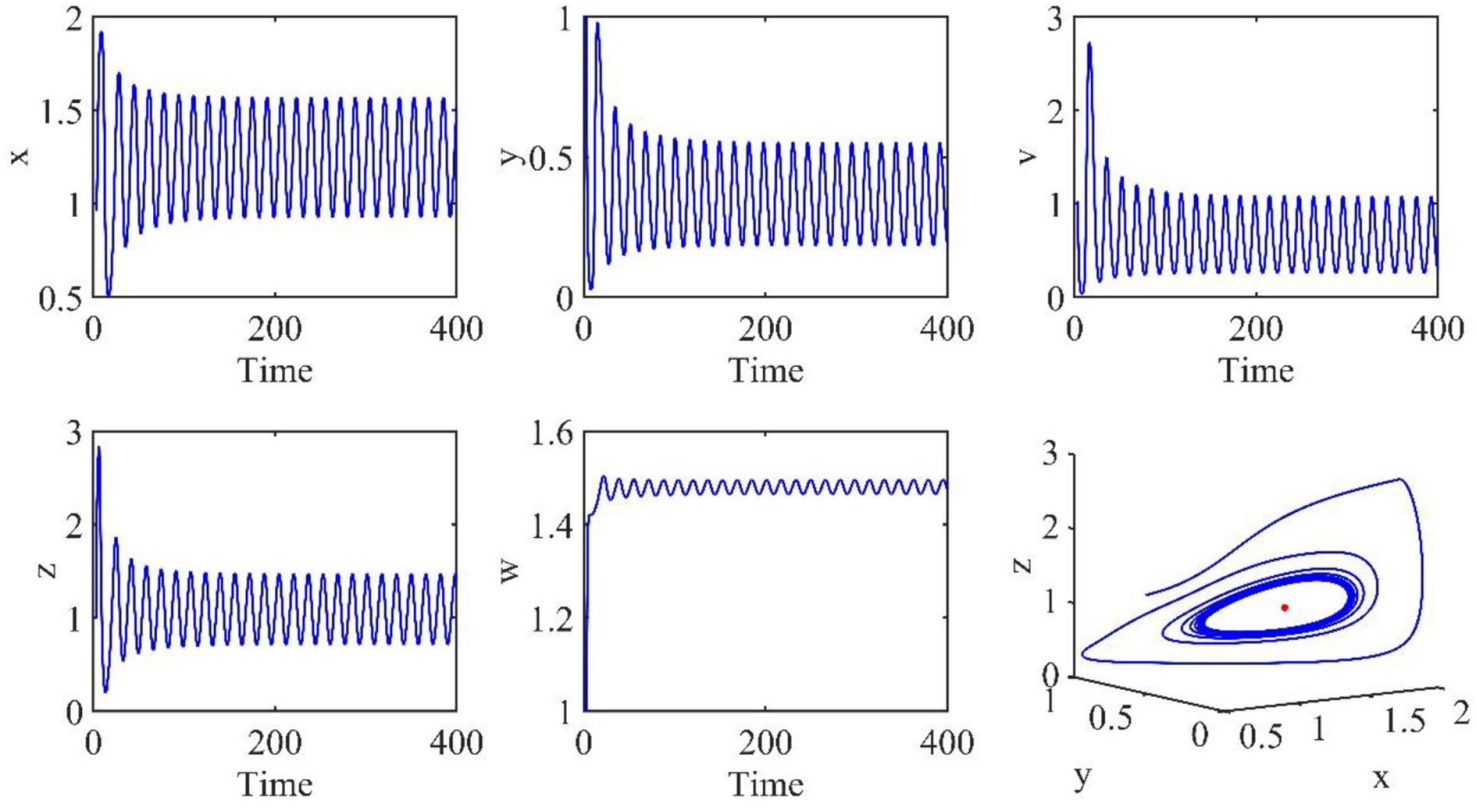
Dynamic change trend of uninfected cells (*x*), infected cells (*y*), free viruses (*v*), CTLs (*z*), ALT (*w*), and phase portraits of the xyz-space with τ = 3.2.

**Figure 5:**
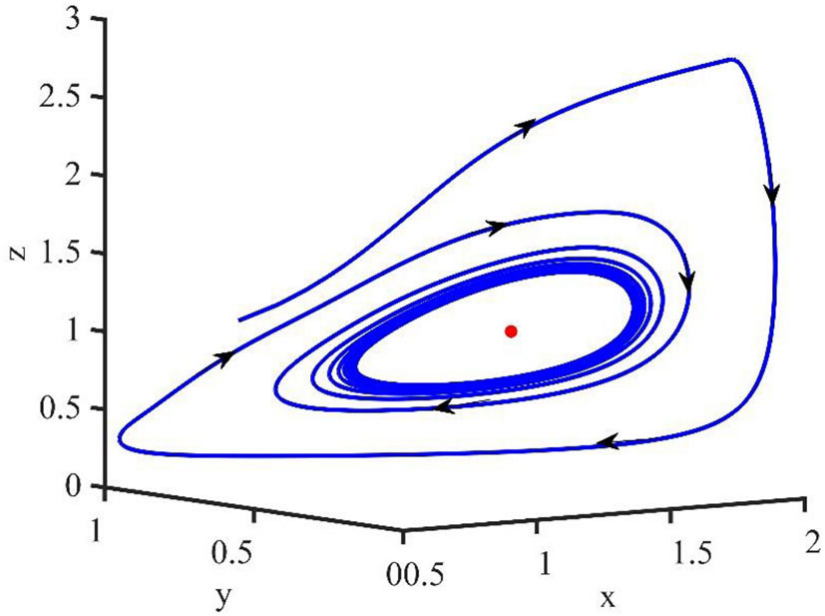
Phase portraits of the xyz-space with τ = 3.2.

**Figure 6:**
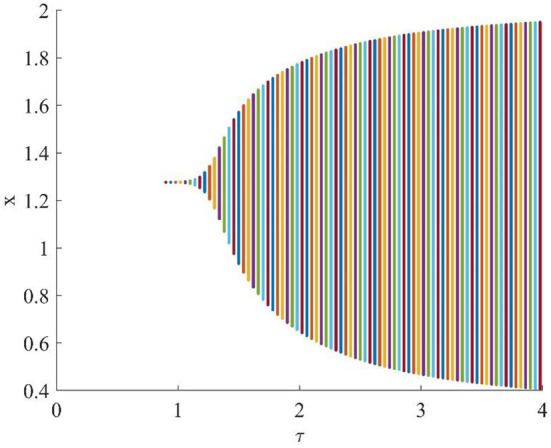
One parameter bifurcation diagram with respect to τ.

Figure 4 shows that when τ = 3.2, the uninfected cells (*x*), infected cells (*y*), free viruses (*v*), CTLs (*z*), and ALT (*w*) oscillate periodically around the infection equilibrium *E*_1_. Figure 5 shows the phase portraits of the uninfected cell–infected cell–free virus space, and when τ = 3.2, the phase portraits are a stable limit cycle which is around the infection equilibrium *E*_1_. The bifurcation diagram (Figure 6) shows that the stability of infection equilibrium *E*_1_ changes at τ = 1.2.

### Simulation of phase portraits with different orders

**Figure 7:**
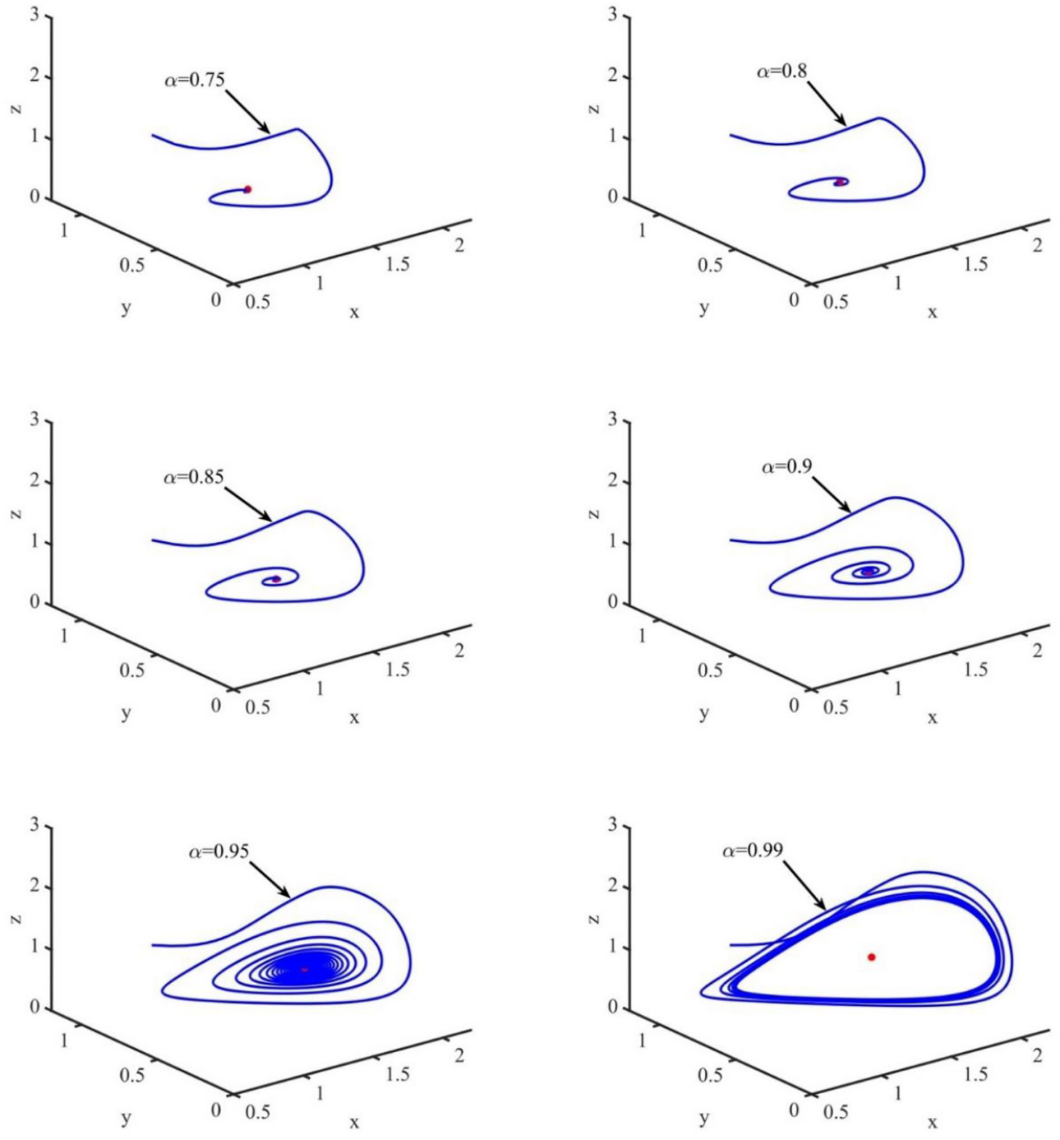
Phase portraits of the xyz-space with τ = 1.2 and α = 0.75, α = 0.80, α = 0.85, α = 0.90, and α = 0.99.

In this section, the phase portraits with different orders are studied by using the method of numerical simulation. The initial order is α = 0.75, and with step of 0.05, the order increases to 0.99. The phase portraits also used the uninfected cell–infected cell–free virus space. As shown in Figure 7, when τ = 1.2 and the order (α) increases from 0.75 to 0.99, the volume of the phase portraits becomes bigger and the phase portraits become more complicated. Furthermore, the numerical simulations indicated that when the order increases from 0.75 to 0.95, the uninfected cell–infected cell–free virus space converges to the infection equilibrium *E*_1_. However, when α = 0.99, the phase portrait is a stable limit cycle, which is around the infection equilibrium *E*_1_. This indicated that the order can significantly affect the stability of the system.

## Conclusion and discussion

In this study, a fractional differential model of HBV infection with time delay and logistic proliferation was proposed in order to better understand the infection mechanism and realize the infection progression. First, the infection-free equilibrium, infection equilibrium, and the basic reproduction number were computed. In epidemiology, *R*_0_ is considered the most important parameter, which provides an insight into how the disease spreads and helps us understand how to control the disease. Therefore, we proved that if the basic reproduction number $R_{0}=\frac{b^{\alpha }k^{\alpha }x_{0}}{a^{\alpha }\varepsilon^{\alpha }}<1$, the infection-free equilibrium (*E*_0_) is locally asymptotically stable, which indicated that if the basic reproduction number *R*_0_ < 1 can be controlled in patients, hepatitis B will disappear. Similarly, the stability analysis of the infection-free equilibrium (*E*_1_) was discussed. In addition, the Hopf bifurcation of the infection equilibrium was studied at the theoretical level. Furthermore, sensitivity was analyzed to screen out the parameters that can significantly affect the basic reproduction number in our model. The results indicated that decreasing the infection rate (*b*), production rate of free viruses (*k*) and production rate of uninfected cells (ξ) can significantly decrease the basic reproduction number (*R*_0_). Similarly, increasing the death rate of infected cells (*a*), the death rate of free viruses (ε) and he death rate of uninfected cells (*d*) can also decrease the basic reproduction number (*R*_0_). Therefore, in order to keep *R*_0_ < 1, the patient can decrease parameters *b*, *k*, and ξ or increase *a*, ε, and *d* to achieve the purpose of treatment.

In order to verify the accuracy of the aforementioned theoretical analysis, the numerical simulations were carried out. The simulation results showed that when *R*_0_ < 1 and τ < 1.2, the infection-free equilibrium *E*_0_ is asymptotically stable, which indicates that the disease will disappear. When *R*_1_ > 1 and τ < 1.2, the infection equilibrium *E*_1_ is asymptotically stable, which indicates that the disease could be mitigated and will lead to a lower infectious class over a period. However, with the increase in τ, the uninfected cells, infected cells, free viruses, CTL levels, and ALT levels oscillate periodically around the infection equilibrium *E*_1_, and the phase portrait is a stable limit cycle, which around the infection equilibrium *E*_1_ indicate that the disease would be out of control. Furthermore, the simulations also indicated that the order can significantly affect the stability of the system. For example, if the order is in the range of 0.75–0.95, the phase portraits converge to the infection equilibrium *E*_1_, and when α = 0.99, the phase portrait is a stable limit cycle.

Therefore, time delay and fractional order are necessary factors that should be considered in modeling HBV infection and for researching dynamic characteristics. Although the process of HBV infection is more complicated than is established in this study, we believe that the model and analysis can play an important role in improving the HBV treatment regimen.

## Data availability statement

The original contributions presented in the study are included in the article/supplementary material, further inquiries can be directed to the corresponding authors.

## Author contributions

DS: conceptualization, methodology, software, and writing—reviewing and editing. JL: software, methodology, and writing. XS: supervision, writing—review editing. GP: writing—review editing. All authors contributed to the article and approved the submitted version.

## Funding

The work is supported by The National Natural Science Foundation of China (62103287 and 62003071), Basic Research General Project of Shenzhen (JCYJ20210324103209026), PhD Basic Research Initiation Project (RCBS20200714114856171), and Clinical Research Project of Shenzhen Second People's Hospital (20213357007).

## Conflict of interest

The authors declare that the research was conducted in the absence of any commercial or financial relationships that could be construed as a potential conflict of interest.

## Publisher's note

All claims expressed in this article are solely those of the authors and do not necessarily represent those of their affiliated organizations, or those of the publisher, the editors and the reviewers. Any product that may be evaluated in this article, or claim that may be made by its manufacturer, is not guaranteed or endorsed by the publisher.
